# Feasibility and Long-Term Compliance to Continuous Positive Airway Pressure Treatment in Adults With Down Syndrome, a Genetic Form of Alzheimer’s Disease

**DOI:** 10.3389/fnins.2022.838412

**Published:** 2022-03-30

**Authors:** Sandra Giménez, Ariadna Farre, Fátima Morente, Laura Videla, Marta Gutiérrez, Susana Clos, Ana Fernández, Marta Blanco, Miren Altuna, Jordi Pegueroles, Amparo Asensio, Bessy Benejam, Mar Batista, Isabel Barroeta, Ana Fortuna, Juan Fortea, Mercedes Mayos

**Affiliations:** ^1^Multidisciplinary Sleep Unit, Respiratory Department, Hospital de la Santa Creu i Sant Pau, Biomedical Research Institute Sant Pau, Barcelona, Spain; ^2^Sant Pau Memory Unit, Department of Neurology, Hospital de la Santa Creu i Sant Pau, Biomedical Research Institute Sant Pau, Universitat Autònoma de Barcelona, Barcelona, Spain; ^3^Center of Biomedical Investigation Network for Neurodegenerative Diseases (CIBERNED), Madrid, Spain; ^4^Barcelona Down Medical Center, Fundació Catalana de Síndrome de Down, Barcelona, Spain; ^5^Centro de Investigación Biomédica en Red de Enfermedades Respiratorias (CIBERNED), Madrid, Spain

**Keywords:** obstructive sleep apnea, CPAP compliance, Down syndrome, Alzheimer’s disease, sleep

## Abstract

**Background:**

Down syndrome (DS) is a genetic form of Alzheimer’s disease (AD) with a high prevalence of obstructive sleep apnea (OSA). These characteristics place the DS population as an optimal model to study the relationship between sleep and AD and to design clinical trials of preventive sleep therapies for AD. Regrettably, OSA treatment with continuous positive airway pressure (CPAP) is often neglected in adults with DS. In both clinical practice and research trials, it is usually presumed that these patients will not adapt to or tolerate the therapy.

**Study Objective:**

We aimed to evaluate the feasibility and long-term CPAP compliance in this population and their capacity to be enrolled in CPAP research studies.

**Methods:**

We prospectively compared the CPAP compliance of 17 OSA patients with DS and 19 age and sex matched OSA euploid patients. CPAP management and follow-up schedules were prescribed according to the habitual clinical practice. We compared group differences in tolerance, objective, and subjective hours of nightly CPAP usage at the 1st, 3rd, 6th, 12th, 24th, and 36th month visits. Good compliance was defined as at least 4 h use per night. We also investigated predictive factors of long-term CPAP compliance.

**Results:**

The percentage of DS subjects with good CPAP compliance (81.2 vs. 78.9%) and the objective CPAP use (5 vs. 6 h, *p* = 0.92) did not differ from the control group (CG). Subjective CPAP compliance was significantly higher in OSA patients with DS than in controls in all the follow-up visits (8 vs. 6.75 h, *p* = 0.001). The DS group had a significantly higher number of visits (9 vs. 5; *p* = 0.021) and mask changes (2.5 vs. 2; *p* = 0.05) than controls. Objective hours of CPAP use at the first follow-up visit predicted long-term CPAP compliance (*p* < 0.005).

**Conclusion:**

CPAP treatment is feasible and has good long-term compliance in OSA patients with DS. It should be recommended to improve health and prevent comorbidities. The DS population is indeed suitable to participate in longitudinal preventive sleep clinical trials for AD.

## Introduction

Down syndrome (DS) is a genetic form of Alzheimer’s disease (AD) with a long and predictable preclinical phase similar to that in the sporadic and autosomal dominant form (Fortea et al., 2020, 2021). The triplication of the amyloid precursor protein (APP) located in chromosome 21 increases the production of amyloid beta (Aβ). The lifetime risk for early-onset Alzheimer’s disease is over 90% (Ballard et al., 2016).

The extra copy of chromosome 21 is also associated with several morphological and functional abnormalities, that among others narrow the upper airway and predispose the DS population to obstructive sleep apnea (OSA) from infancy (Simson et al., 2018). With aging, this risk increases, partly due to the development of hypothyroidism and obesity (Lal et al., 2015). The prevalence of OSA in adults with DS ranges between 65 and 89% (Trois et al., 2009; Giménez et al., 2018, 2021; Landete et al., 2020), which represents that OSA is at least five times more common in DS than in the general population (Heinzer et al., 2015).

Obstructive sleep apnea is a sleep breathing disorder characterized by episodes of upper airway occlusion, that entails chronic intermittent hypoxemia, hypercapnia, metabolic abnormalities, sleep fragmentation, and cerebral hypoperfusion (Lévy et al., 2015). OSA is independently associated with excessive daytime sleepiness, cognitive impairment, depression, cardiovascular disease, and all-cause mortality (Gottlieb and Punjabi, 2020). Indeed, increasing evidence supports a pathophysiological relationship between OSA and Alzheimer’s disease, as demonstrated by links between mentioned mediating OSA factors and AD biomarkers of neurodegeneration (Sharma et al., 2018; Bubu et al., 2019, 2020).

Whereas AD does not yet have an effective treatment, some of the pathologic mechanisms in OSA can be reverted by OSA treatment helping to prevent cognitive decline (Mullins et al., 2020). OSA, thus, may be an important modifiable risk factor for AD prevention.

Continuous positive airway pressure (CPAP) is considered the first line treatment for OSA (Patil et al., 2019). In the general population CPAP reduces sleepiness, the risk of cardiovascular comorbidities, and improves daytime functioning (Marin et al., 2005). In patients with mild cognitive impairment and AD, CPAP treatment also improved cognition and delayed the age of AD diagnosis (Osorio et al., 2015; Liguori et al., 2017). Further OSA longitudinal studies examining the possible protective effect of CPAP treatment on AD biomarkers in the preclinical stages of AD are required to study the relationship between OSA and AD (Liguori et al., 2021). To advance in this knowledge, it has been suggested that further complementary research should move into different populations and on to phase III clinical trials (Bubu et al., 2020; Mullins et al., 2020).

In the population with DS, as in the general population, OSA may impair cognitive function and accelerate the progression to AD dementia (Cody et al., 2019; Giménez et al., 2021). The elevated risk of both AD and OSA in adults with DS makes this population ideally an optimal model not only to study this relationship, but also to conduct clinical trials of preventive sleep therapies for AD.

Obstructive sleep apnea treatment with CPAP, however, is not usually proposed in adults with DS. Both caregivers and physicians often presume that these patients will not tolerate CPAP and that additional complex interventions are needed to ensure compliance with the treatment. This may be reinforced by the limited data about CPAP treatment in adults with OSA and DS, and by the absence of objective studies evaluating the feasibility, efficacy, and long-term compliance of CPAP therapy in this population (Hill et al., 2020; Landete et al., 2020).

In this study we aimed to evaluate the feasibility and long-term compliance of CPAP treatment in adults with OSA and DS when using the same clinical protocol of CPAP management routinely used in the general population. We also investigated the predictive factors associated with long-term CPAP compliance.

## Materials and Methods

### Study Population

Neuroimaging Initiative (DABNI), a research health program to screen for Alzheimer’s dementia (AD) in adults with DS that includes a complete sleep study (Alcolea et al., 2019). The patient’s characteristics and the sleep study protocol procedures and results have been described in detail elsewhere (Giménez et al., 2018). Briefly, between September 2014 and June 2016, 47 community-based participants with DS not selected or referred for sleep disorders underwent a polysomnography (PSG), of whom 78% had OSA. For the present CPAP compliance study, we prospectively collected follow-up data from those DS participants with OSA and in whom CPAP therapy was proposed. Indications of CPAP treatment were based on clinical criteria (Lloberes et al., 2011).

We also retrospectively recruited a control group (CG) of 19 euploid OSA patients from the general population treated with CPAP and matched for age, sex, and body mass index (BMI) with the DS group. The study was performed in the multidisciplinary Sleep Unit of the Hospital of Sant Pau in Barcelona, in collaboration with the Alzheimer Down Unit at the same hospital. The local ethics committee following the principles stated approved the study in accordance with the Declaration of Helsinki. Written informed consent was obtained from all participants and from legal caregivers with agreement from the DS participants.

All data were obtained according to good clinical practice (GCP) guidelines.

### Procedures

Continuous positive airway pressure was prescribed following the same routine procedures that are conducted in our sleep unit for OSA patients. These include accurate pressure setting, patient education, and regular follow-up visits. Any additional CPAP intervention was introduced for DS patients. Briefly, after diagnosis of OSA, all patients had a consultation where the sleep physician explained OSA and its consequences including the indication, rationale, and potential benefits of OSA treatment with CPAP, and the need to perform another PSG for CPAP titration.

#### Continuous Positive Airway Pressure Titration

With the intention of optimizing treatment tolerance, CPAP adaptation was performed in the same day of in-hospital PSG titration. In the presence of the caregiver, qualified technical staff explained to the patient in detail the indication, objectives, and potential discomfort that may be experienced during the titration.

The patient was accommodated in bed, and the most appropriate and comfortable facemask was chosen for each case according to facial morphology. When the patient felt comfortable, low-pressured air was started (at 4 cmH_2_O) and slowly increased to medium pressures. The patients were then invited to try to nap for about 30 min. After completing this procedure, the patients were subjected to the titration study that same night. All patients underwent a night-time CPAP titration using conventional PSG at the sleep unit with manual titration, according to the American Academy of Sleep Medicine recommendations (Kushida et al., 2008). In the morning, before leaving, an explanatory CPAP brochure was given to the caregivers, which included a user manual and a telephone contact number with the CPAP specialist nurse.

#### Continuous Positive Airway Pressure Follow up Visits

After CPAP prescription, all patients attended follow-up CPAP visits conducted by an experienced CPAP specialist. The CPAP follow-up visits were carried out as performed in our clinical practice: at months 1, 3, 6, and 12, and then annually or whenever required by the patient or caregivers. At each follow-up visit, compliance, tolerance, and autonomy with the CPAP device were checked. Any concerns with the interface and/or CPAP device were also addressed and changed, if necessary. The autonomy was checked by asking the patient to put the mask on and to turn the device on. In each follow-up visit, the importance of the CPAP use was reinforced, and patients were encouraged to keep the mask on every sleeping hour.

The CG followed the same protocol, except for CPAP titration, which was performed by conventional PSG or automatic CPAP in the sleep lab or at home following a validated protocol (Masa et al., 2004). The CPAP explanatory brochure, with a user manual and a telephone contact number with the CPAP specialist nurse, was also provided to the control participants.

### Data Collection/Endpoints

We collected baseline clinical and demographic data in all patients. Compliance CPAP variables were systematically recorded prospectively including:

-*Objective CPAP compliance*: Hours of nightly usage at months 1, 3, 6, 12, 24, and 36 months after CPAP initiation was obtained by reading the time-counter of the CPAP device. The download data at each visit corresponds to the average usage in the preceding time interval since the previous visit. The primary outcome variable in this study was the number of hours of CPAP nightly use at follow-up and was calculated through two variables: hours of CPAP usage at last visit and average hours of CPAP use across all visits. Good compliance to the CPAP treatment was considered as use of CPAP >4 h per night, and non-compliance was considered as use of CPAP ≤4 h of use per night (Patil et al., 2019).
- *Subjective CPAP compliance*: Estimated as the nightly number of hours of CPAP use as reported by the patients (or by the caregivers in the case of DS patients), at each follow-up visit. Final subjective compliance was calculated as the reported number of hours of CPAP nightly use at follow-up and was also calculated through two variables: reported hours of CPAP usage at last visit and reported average hours of CPAP use across all visits.
-*Tolerance* parameters concerning the degree of comfort with the mask, the presence of any side effects, such as nasal stuffiness, skin irritation, or eye puffiness were systematically recorded. Any concern with the interface and/or CPAP device were also addressed and changed if necessary.
-*The degree of autonomy* of the patient using the CPAP device was noted as high if the patient could put on and remove the mask alone, and low if the CPAP mask assistance from caregivers was always required.

Dropouts were defined as patients who were prescribed CPAP but never started the treatment and/or never attended the follow-up visits.

The total number of additional visits within the planned interval schedule was also included. We assessed the factors associated with objective CPAP compliance at the end of the follow up period, including: (1) socio-demographic characteristics of the patients: age, gender, level of intellectual disability or social support, (2) baseline OSA severity: apnea–hypopnea index (AHI), cumulative time percentage with Spo2 <90% (CT90), severity of somnolence measured by Epworth Sleepiness Scale (ESS), (3) CPAP variables: CPAP pressure, interface type (nasal, oronasal, or nasal pillows), number of visits, side effects, and number of mask changes.

### Statistical Analyses

Demographic characteristics were summarized using the median and interquantile range (IQR) for continuous variables and percentages for categorical variables. Mann–Whitney test was used to test differences between groups. Additionally, Cliff’s delta was computed to test the effect size between groups. To test the evolution of CPAP compliance between groups over time we performed linear mixed-effects (LMEs) models using the maximum likelihood method for the change in CPAP over time. We included age, sex, group, time from baseline, and the interaction between time and group as fixed effects and intercept and time from baseline were included as subject-specific random effects.

Continuous positive airway pressure predictive factors were evaluated by Spearman’s rank correlation analyses. The time to achieve good compliance was taken as the visit that was significantly above the compliance threshold. All plots and statistical analyses were performed using R statistical software (v 3.6.3). A *p*-value of less than 0.05 was considered statistically significant and a Cliff’s delta (*d*) of more than 0.47 was considered a large effect size.

## Results

Continuous positive airway pressure treatment was proposed for 17 patients with DS during the study period. All accepted and completed a PSG and CPAP titration in the hospital. One OSA patient with DS refused to start CPAP treatment and dropped out before the first follow-up visit.

The demographic characteristics of OSA patients with DS and the CG are summarized in Table 1. In the DS group, median age was 45 years (range 35–52), the majority were men (12, 70.6%), and had severe OSA (14, 82.3%). Only three had moderate OSA (17.65%). There were no differences in age, sex, BMI, daytime somnolence (ESS), and AHI or CPAP pressure between OSA patients with DS and the CG. Five control subjects had a history of hypertension (HTA), but none in the DS group.

**TABLE 1:** The demographic and baseline characteristics of OSA patients with Down syndrome and OSA patients from the control group.

	DS patients (*n* = 17)	Control patients (*n* = 19)	*p*-value
Age (years)	45.0 (35.0–52.0)	47.0 (40.5–51.0)	0.861
Female, *n* (%)	5 (29.4)	4 (21.1)	0.847
BMI (kg/m^2^)	32.5 (28.2–34.6)	33.4 (30.35–35.75)	0.228
ESS	7.0 (4.0–13.0)	12.0 (7.0–16.0)	0.131
AHI (events/h)	48.7 (36.5–74.4)	63.0 (36.5–65.65)	0.788
CPAP pressure (cmH_2_0)	8.0 (7.0–9.0)	9.0 (7.5–10.0)	0.192
Hypertension *n* (%)	0 (0)	5 (26.3)	0.072
Dyslipidemia	0 (0)	4 (21.1)	0.140
Diabetes mellitus	1 (5.9)	3 (15.8)	0.680
Cardiopathy *n* (%)	5 (29.4)	2 (10.5)	0.314

*Descriptive data are presented as n: number of subjects (percentages) or median and interquartile range (IQR).*

*DS, Down syndrome; BMI, body mass index; ESS, Epworth Sleepiness Scale; AHI, apnea–hypopnea index; CPAP, continuous positive airway pressure.*

At the end of the study period, no significant differences were observed in the median objective CPAP use between groups (Table 2). Thirteen DS patients (81.2%) had good CPAP compliance. From the three non-compliant OSA patients with DS, one patient remained on CPAP therapy although insufficiently, and two patients dropped out after 1 month. In the CG, all patients started CPAP treatment and 15 patients (78.95%) had good compliance. From the four non-compliant participants, one patient dropped out after the first follow-up visit. There was no difference in hours of CPAP use between the DS group (5.60 h, range 4.50–6.80) and controls (5.67 h, range 4.92–6.00) within the subgroup of good compliance patients (*p* = 0.963). In the CG there were no significant differences in the average of objective CPAP compliance (*p* = 0.96) neither in subjective CPAP compliance (z = 0.206) between patients with and without hypertension.

**TABLE 2:** Continuous positive airway pressure follow up measurements.

	DS patients (*n* = 16)	Control patients (*n* = 19)	*p*-value
Objective CPAP use (last visit)	5.00 (4.00–7.25)	6.00 (5.0–6.0)	0.920
Objective CPAP use (average)	4.86 (4.00–6.65)	5.17 (4.40–5.90)	0.934
Subjective CPAP use (last visit)	8.0 (7.75–9.00)	7.00 (6.50–7.0)	0.001
Subjective CPAP use (average)	8.0 (7.12–8.50)	6.75 (6.12–7.0)	0.001
Good compliance (%)	13.0 (81.2)	15.0 (78.9)	1.00
Follow up (months)	37 (29.7–39.0)	25.0 (1.5–34.0)	0.123
Number of visits	9.0 (7.0–13.0)	5.0 (4.0–7.70)	0.021
Number of mask changes	2.50 (1.75–4.0)	2.0 (0.5–2.0)	0.050

*Descriptive data are presented as n: number of subjects (percentages) or median and interquartile range (IQR).*

*CPAP, continuous positive airway pressure.*

**FIGURE 1:**
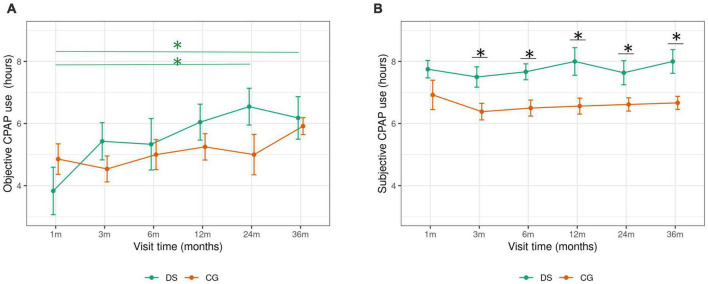
Objective and subjective CPAP compliance follow up in OSA patients with OS and CG. DS, Down syndrome; CG, control group; CPAP, continuous positive airway pressure; m, month. Objective and subjective trajectory of CPAP compliance is represented by the mean (+SE) nightly CPAP use at each follow up visits (1–36 months). *Significant differences (*p* < 0.05) in CPAP use across visits **(A)**, and between groups **(B)** are indicated.

When assessing the LMEs analysis, in both groups, the duration of objective CPAP use increased gradually in the follow-up (results from the LME analyses are shown in Supplementary Table 1). In OSA patients with DS, there was a significant increase in CPAP use from the first month visit (4 h, range 0.82–6.25) compared to the 24-month visit (7 h, range 5.50–7.50, *p* = 0.05) and between the first and the 36-month visit (6 h, range 5.0–8, *p* = 0.05) (Figure 1A). In OSA patients with DS good CPAP compliance was achieved after 3 months of treatment. No significant differences in CPAP use among visits were observed in the control patients. There was no significant difference in CPAP use across visits between DS and controls.

At the end of the study period, the percentage of subjects with good CPAP compliance was comparable in both groups. There was a tendency toward a lower frequency of compliance in the DS group compared to the controls (56 vs. 86%, *p* = 0.084) in the first month follow-up visit only. In the DS group a tendency toward increased compliance was observed between the 1-month and 3-month visits (56 vs. 86%, *p* = 0.089), and between the 1-month and both 24- and 36-month visits (56 vs. 90%, *p* = 0.061).

**FIGURE 2:**
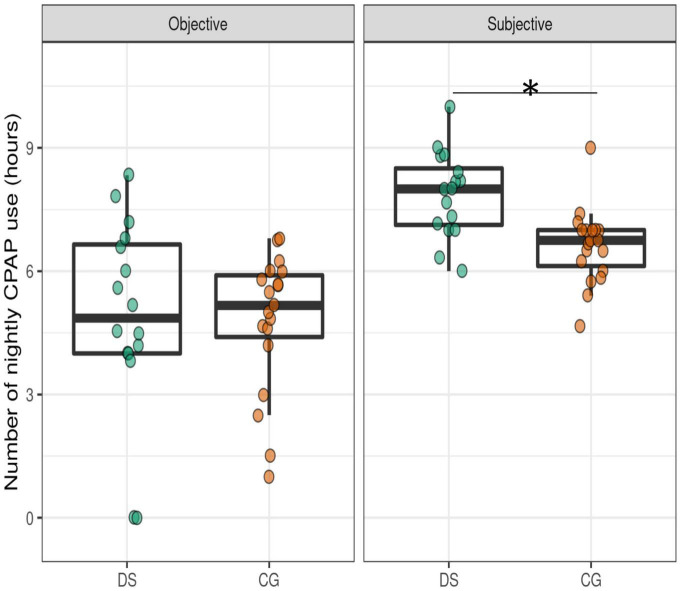
Objective and subjective CPAP use in OSA patients with DS and CG. DS, Down syndrome; CG, control group; CPAP, continuous positive airway pressure. Boxplots illustrate the distribution of nightly CPAP use showing the individual data points and the median average use. *Significant differences (*p* < 0.05) between groups are indicated.

In average, subjective CPAP use was higher than the objective use in both groups (Figure 2). OSA patients with DS reported significantly higher subjective compliance compared to control participants, on the average CPAP use at the end of the study period (Table 2), and in every follow up visit, except the first month follow up visit. In both groups, a good CPAP compliance was already reported from the first follow up visit (Figure 1B). There were no changes in subjective CPAP use across visits in the follow-up period in the DS group or in the CG.

Obstructive sleep apnea patients with DS had a significantly higher median number of visits compared to the CG (Table 2).

Fourteen OSA patients with DS (87.5%) used a nasal mask and 2 an oronasal mask. The most common side effects were due to problems with the interface (93.75%) and nasal congestion (25%). The number of mask changes was significantly increased in patients with DS vs. controls. Four patients (25%) were highly autonomous in the management of CPAP, others needed help from their caregivers. Only one OSA patients with DS lived in a group home and did not show good compliance.

Objective hours of CPAP use at the first follow-up visit were the only predictive factors of good CPAP compliance at 3 years for both the DS (*p* < 0.001, rho = 0.78) and control (*p* = 0.004, rho = 0.71) groups.

In the DS group, better CPAP compliance was not significantly but was nominally associated with a major number of visits (*p* = 0.28; *d* = 0.41) and higher Epworth scores (*p* = 0.2; *d* = 0.56).

In the CG, an increased number of visits (*p* = 0.03; *d* = 0.7), an absence of cardiopathy (*p* = 0.048; *d* = 0.5), a higher baseline CT90 (*p* = 0.044; *d* = 0.53), and a trend for an association with higher IAH (*p* = 0.06; *d* = 0.62) were associated with better long-term CPAP compliance.

## Discussion

This study shows that CPAP for OSA treatment is feasible in adults with Down syndrome, with good and long-term compliance applying the same clinical practice of CPAP management than in the general population. Besides the clinical relevance of the data, it also demonstrates the capacity of adults with DS to participate in CPAP clinical research studies.

Four hours cut-off point is the most accepted criterion to differentiate between bad and good compliance to CPAP treatment (Patil et al., 2019). In the general population, the percentage of good CPAP compliance ranges from 28 to 83% (Sawyer et al., 2011), with higher rates at the beginning of the treatment (Mehrtash et al., 2019), and decreases in adherence in longer follow-up periods (Rotenberg et al., 2016; Cistulli et al., 2019). In the present study, CPAP compliance in OSA adults with DS was lower in the first month, but gradually and significantly increased from the first to the last visit, in contrast to controls (Rotenberg et al., 2016; Baratta et al., 2018; Cistulli et al., 2019). After 3 years of follow-up, 81% of OSA patients with DS were considered to have good CPAP compliance, with nightly CPAP use of 5 h, a compliance that is comparable to that of the CG.

There are scarce and heterogeneous data in the literature relating to the suitability of CPAP treatment in adults with DS. Good CPAP compliance has been reported in 62.5% of subjects with DS after 1 year of treatment (Trois et al., 2009), in 60% of subjects after an intensive 6 months of CPAP training protocol (Weaver and Grunstein, 2008), and in 79% of adults with DS symptomatic for OSA (Landete et al., 2020). Insufficient CPAP compliance was reported in a prospective CPAP randomized study after 1 year of follow-up (Hill et al., 2020). In most of these studies, however, methodological data regarding compliance or follow-up measurements were not provided. Our study substantially differs from the above studies and adds important new data: (1) participants followed the habitual clinical practice of CPAP management in our sleep unit, (2) the study included substantially longer follow-up with a detailed trajectory of CPAP use, (3) an additional comparison between subjective and objective data was assessed, (4) inclusion of control OSA patients from the general population that had the same clinical follow-up protocol, (5) factors predicting CPAP compliance were explored, and (6) the study was not subject to selection bias since our patients were not selected due to sleep complaints.

We investigated several patient, sociodemographic, and disease factors that have been assessed in relation to CPAP compliance (Shapiro and Shapiro, 2010; Sawyer et al., 2011; Luijks et al., 2017; Gottlieb and Punjabi, 2020).

In the general population, the patient’s perception of OSA symptoms, risks, benefits of the therapy, and measures of self-efficacy increase self-motivation to CPAP use (Shapiro and Shapiro, 2010; Luijks et al., 2017). In subjects with DS, the management and supervision of CPAP depends mainly on OSA education and motivation of their caregivers. Caregivers were concerned that the effectiveness of the therapy relies on CPAP acceptance and compliance (Griffin et al., 2013; Luijks et al., 2017), and early side effects and troubleshooting were quickly communicated during the follow-up period. OSA patients with DS, probably due to their narrow nasal passages, presented more mask discomfort, that resulted in more mask changes than in controls (Aloia et al., 2005). Additional follow-up visits to correct these issues resulted in increased CPAP compliance (Aloia et al., 2005; Griffin et al., 2013). Another finding of our study regarding caregivers is that they overestimated subjective CPAP use more than the discrepancy reported in the general population (Rauscher et al., 1993). This misperception was already present in the first visit, despite insufficient objective compliance in this period, but contributed to their favorable opinion of the treatment.

Our results are clinically relevant as they provide evidence that CPAP treatment is not only feasible in OSA adults with DS, but also perfectly integrated in patients and caregivers’ routines, and can be sustained for extended periods without major changes.

Disease factors, such as baseline OSA severity and excessive daytime somnolence (ESD), were not associated with better long-term CPAP compliance in OSA patients with DS, in contrast to controls. In the general population, it is likely that the relief of these OSA symptoms increases motivation to endure treatment (Griffin et al., 2013; Jacobsen et al., 2017), despite data being inconclusive (Kohler et al., 2010). In the intellectual disability population, it has been proposed that other disease factors, such as improvement in sleep quality, may in part explain the high CPAP tolerance observed (Campos-Rodriguez et al., 2005; Weaver and Grunstein, 2008). CPAP compliance may vary with age, with younger subjects presenting worse compliance (Kang et al., 2019; Giménez et al., 2021). Maybe middle-aged patients actually represent the milder end of the OSA severity spectrum, with better CPAP compliance. On the other hand, most adults with DS present OSA already from infancy, which could lead to a progressive clinical adaptation to adverse symptomatology of chronic OSA. Further polysomnography sleep studies are needed to evaluate objective sleep changes after CPAP treatment to assess improvements in sleep quality and their relation to CPAP compliance across ages.

Objective hours of CPAP use at the first follow-up was the only predictor of long-term use in both groups, thus highlighting the importance of early CPAP compliance in the DS population, as has been reported in the general population (Kang et al., 2019; Mehrtash et al., 2019). This is extremely important since, contrary to the general population, CPAP treatment in the DS population is not habitually initiated to treat acute OSA symptoms to prevent cardiovascular problems or to reduce risk of accidents and, therefore, the perception of the urgency and the need for CPAP treatment can be low (Weaver and Grunstein, 2008).

Life expectancy in the population with DS has increased, and accordingly, comorbidities associated with aging, such as OSA and Alzheimer’s disease. Further studies are need to determine the efficacy of CPAP treatment in minimized additional cognitive impact and decelerate the progression to symptomatic (Lal et al., 2015; Simson et al., 2018). Unfortunately, adults with Down syndrome have not been included in preventive AD clinical trials. Ethic challenges and concerns about the feasibility of completing all assessments compared with those done in the general population are the main reasons. Luckily, the exponential increasing research in AD in the DS population is rapidly reversing this scenario (Hendrix et al., 2020; Fortea et al., 2021). Briefly, the Down syndrome population has (1) an ultra-high risk for developing symptomatic Alzheimer’s disease with a long preclinical phase in which biomarkers follow a predictable order of changes over more than 2 decades, (2) a high OSA prevalence, (3) a higher prevalence than autosomal dominant Alzheimer’s disease, and (4) a more homogeneous pathophysiology than those with sporadic AD. These characteristics place people with Down syndrome as an optimal population to be included in studies to evaluate the relationship between OSA and AD, and in clinical trials of preventive sleep therapies for AD. Moreover, given the similarities between both sporadic and autosomal dominant AD and AD in individuals with Down syndrome, results and therapies of these sleep studies could be beneficial for all these groups.

This study has some limitations. Despite being one of the largest studies on CPAP compliance, it has a relatively small sample size, which may have limited the power to establish significant differences in some variables. Most of the participants were middle-aged adults. Outside these ages ranges, results in CPAP compliance may differ. DS participants came from a population-based health plan design to screen for AD in DS, reflecting the population of adults with DS in Catalonia and the CG was recruited during clinical practice at a sleep clinic. This might have increased the motivation to CPAP treatment in the controls, but despite this fact, CPAP compliance in the DS population was comparable. Moreover, due to the small sample size and the variable levels of intellectual disability associated with DS, changes in cognitive function after CPAP treatment could not be provided. Further larger studies are needed to assess the efficacy of CPAP treatment in this population.

In conclusion, our study showed that CPAP treatment is feasible in OSA adults with DS, and with a good long follow-up compliance when adhering to the usual clinical practice of CPAP management that is applied to the general population.

These data underlie that OSA treatment should be recommended and might prevent comorbidities and improve the quality of life in this vulnerable population. It also highlights the capability and willingness of adults with Down syndrome to participate in CPAP research studies of preventive sleep therapies for AD.

## Disclosure

Work for this study was performed at Santa Creu i Sant Pau University Hospital in Barcelona. All co-authors have reviewed and agreed on the contents of the manuscript and there is no financial interest to report.

## Data Availability Statement

The raw data supporting the conclusions of this article will be made available by the authors, without undue reservation.

## Ethics Statement

The studies involving human participants were reviewed and approved by the Clinical Research Ethics Committee (CEIC), Hospital de la Santa Creu i Sant Pau, Barcelona, Spain. The patients/participants provided their written informed consent to participate in this study.

## Author Contributions

SG and MM developed the study concept and design. AFa, FM, LV, MG, SC, AFe, MBl, MA, AA, BB, MBa, IB, and AFo acquired the data. SG, JP, and MM analyzed and interpreted the data, did the statistical analysis, and drafted the manuscript. All authors revised and edited the manuscript and critically revised it for important intellectual content.

## Conflict of Interest

The authors declare that the research was conducted in the absence of any commercial or financial relationships that could be construed as a potential conflict of interest.

## Publisher’s Note

All claims expressed in this article are solely those of the authors and do not necessarily represent those of their affiliated organizations, or those of the publisher, the editors and the reviewers. Any product that may be evaluated in this article, or claim that may be made by its manufacturer, is not guaranteed or endorsed by the publisher.

## Abbreviations

DS: Down syndrome
AD: Alzheimer’s disease
PSG: nocturnal polysomnography
AHI: apnea–hypopnea index
OSA: obstructive sleep apnea
CPAP: continuous positive airway pressure
ESS: Epworth Sleepiness Scale.
