# Supplementary material for: Feasibility and Long-Term Compliance to Continuous Positive Airway Pressure Treatment in Adults With Down Syndrome, a Genetic Form of Alzheimer’s Disease
Source: Front Neurosci. 2022 Mar 30;16:838412. doi: 10.3389/fnins.2022.838412 (PMC9005794; doi:10.3389/fnins.2022.838412)
Supplement: Supplementary file 1 [file Table_1.DOCX]

**Supplmentary Table 1**: The Estimates, standard error (Std.Error) and T-value for each fixed effect in the linear mixed effects models. Additionally, the variance and standard deviation of the random effects for each subject (intercept and time) are shown.

| **Objective CPAP use** | | | | | | |
| --- | --- | --- | --- | --- | --- | --- |
| Fixed effects: |  |  |  | Random effects for ID: |  |  |
|  | Estimate | Std.Error | t.value |  | Variance | Std.Dev. |
| (Intercept) | 6.3714 | 1.4167 | 4.497 | (Intercept) | 2.3164 | 1.5219 |
| Group | 0.0499 | 0.6600 | 0.076 |  |  |  |
| Time | 0.0480 | 0.0131 | 3.673 | Time | 0.0001 | 0.0101 |
| age | -0.0182 | 0.0282 | -0.643 |  |  |  |
| sex | -0.8671 | 0.6664 | -1.301 |  |  |  |
| Group*Time | -0.0241 | 0.0205 | -1.175 |  |  |  |

| **Subjective CPAP use** | | | | | | |
| --- | --- | --- | --- | --- | --- | --- |
| Fixed effects: |  |  |  | Random effects for ID: |  |  |
|  | Estimate | Std.Error | t.value |  | Variance | Std.Dev. |
| (Intercept) | 9.046956 | 0.78254 | 11.561 | (Intercept) | 0.7668 | 0.8757 |
| Group | -1.1909 | 0.35149 | -3.388 |  |  |  |
| Time | 0.00877 | 0.00776 | 1.130 | Time | 0.0003 | 0.0189 |
| age | -0.01616 | 0.01568 | -1.031 |  |  |  |
| sex | -0.51037 | 0.37488 | -1.361 |  |  |  |
| Group*Time | -0.00258 | 0.01189 | -0.217 |  |  |  |
